# Drug Addiction in Patients With Chronic Schizophrenia and Its Relation With Psychopathology and Impulsiveness

**DOI:** 10.5539/gjhs.v7n7p131

**Published:** 2015-03-27

**Authors:** Somayeh Shokrgozar, Reza Ahmadi, Azadeh Yousefnezhad, Mahbobeh Roshandelrad, Termeh Khosravi, Masuomeh Ellahi, Mahdiyeh Pakdaman, Ameneh Eskandari

**Affiliations:** 1 Department of Psychiatry, Shafa Hospital, School of Medicine, Guilan University of Medical Sciences, Rasht, Iran; 2 Department of Genicology, Azzahra Hospital, School of Medicine, Guilan University of Medical Sciences, Rasht, Iran; 3 Shafa Hospital, School of Medicine, Guilan University of Medical Sciences, Rasht, Iran; 4 Department of Psychiatry, Iran Hospital, School of Medicine, Iran University of Medical Sciences, Tehran, Iran

**Keywords:** Drug Dependence, Chronic Schizophrenia, Impulsiveness

## Abstract

**Background:**

Using drugs is a common affliction in patients with Schizophrenia affecting their increasing death rate. They have to tolerate longer treatment time and staying in hospital and they further show more violence and their living quality decreases. It also seems that this factor is among the influential factors of unsuccessful results in treating these patients.

**Objectives:**

Despite all this, there is little data about drug consumption, psychopathology and demographic information in patients with chronic schizophrenia in Iran. This paper reviews the relation between drug consumption and the mentioned qualities in patients afflicted by chronic Schizophrenia.

**Methods:**

In this cross-sectional study, 100 patients with Schizophrenia were interviewed based on DSM-IV-TR diagnostic parameters and according to a psychiatrist´s views. The severity of psychopathology was evaluated, using PANSS, (SCID-I) DSM-IV and BARRAT.

**Results:**

The results show that in patients with chronic schizophrenia, there is a meaningful relation between cigarette consumption and education, gender, family background and BARRAT. It also has a direct correlation with Attention and Motor. Drug consumption has a meaningful relation with gender and Motor (p< 0.05). But it has no relation with BARRAT. Of the variables having a relation with correlation, cigarette and treatment period factors have a predicting effect for drug consumption.

**Conclusions:**

According to the results, drug and cigarette consumption is high among patients with Chronic Schizophrenia. Common cigarette consumption and its relation with impulsiveness increase, and death rate are the reasons which make us take the needed steps to have these patients quit smoking.

## 1. Introduction

Drug abuse is common in patients with Schizophrenia (Adam, Okan, & Aslı, 2012). Nearly 50% of these patients have the full criteria for both Schizophrenia and drug abuse disorders (Regier et al., 1990). If drug abuse continues, it can cause a decrease in living quality, longer periods in hospital, higher relapse, less treatment adaptation and higher aggressive behavior (Fazel, Langstroem, Hjern, Grann, & Lichtenstein, 2009). The reason behind the high cigarette consumption in Schizophrenia is not completely known yet. The mostly mentioned theory is self-treatment. According to this theory, patients with Schizophrenia use the cigarette smoke to decrease the positive and negative syndrome and improve the recognition function.

It is suggested that smoking improves the side effects of extrapyramidal and further promotes the cognitive side effects of Antipsychotic drugs through decreasing the drugs blood level and increasing the Dopamine activity (Kumari & Postma, 2005). Patients with Schizophrenia abuse alcohol and some other drugs like Nicotine, Cocaine and Cannabis (Winklbaur et al., 2006). These drugs lead to Dopamine activity increase, especially in (Gerdeman et al., 2003). In long drug abuse, Dopamine disorder increases, leading to more disorder in Fronto-Subcortical circuits in patients afflicted by Schizophrenia (Siever et al., 2004). The findings show that patients with co-morbid of drug abuse have higher behavioral impulsiveness compared to healthy people.

Aggression can be divided into affection violence, and fierce. Affection violence includes inimical manner against some threats from the outside world or an internal planned fear. On the other hand, fierce violence has no repentance and the afflicted person has an aggressive manner to fight back and he can have a controlling feeling or can reach a goal (Lejoyeux, Nivoli, Basquin, Petit, Chalvin, & Embouazza, 2013).

In an epidemiologic study of aggressive manner in Schizophrenia, the probability of violence is more, especially if it comes along with hallucination leading to negative feeling (anger, anxiety, sorrow and grief) and if there are no successful strategies to counter act (Cheung et al., 1997; Nolan et al., 2005). Violent manners increase due to severe alcohol abuse or drug intoxication. Drug abuse and alcohol disorders increase violence (Winklbaur et al., 2006).

As there are no exact statistical figures for the drug consumption pattern in patients with Schizophrenia in Iran and because drug abuse in these patients worsens sickness Prognosis and increases impulsiveness, this paper aims to find out the relation between drug abuse, psychopathology severity, impulsiveness, and some Demographic qualities of patients with chronic Schizophrenia. It is assumed that drug abuse and cigarette have a meaningful correlation with positive and negative signals in Schizophrenia which can increase impulsiveness.

## 2. Materials and Methods

The criteria for this study included chronic Schizophrenia diagnosis and the proportional ability to answer the questionnaires. Patients afflicted by acute phase Schizophrenia, mental deficiency, brain diseases like brain tumor, epilepcy, systemic diseases, or with brain operations were excluded.

Based on the previous studies and Cranach’s formula and while considering a=0.05, with the implicit accuracy of 0/5 %, the specimen volume of 100 patients were considered. Of 110 patients, 10 of them were excluded due to specific criteria and the study was done on 100. To have a homogeneous group, just patients with chronic Schizophrenia were chosen. The patients were chosen with an easy and available sampling method.

Before diagnosing Schizophrenia in these people, DSM-IV axis I (I-SCID) (First et al., 1996) and PANSS questionnaire were used by the psychiatrist to give the ultimate decision. All the patients were having the proportional remission of acute diagnoses.

Firstly, the patients´ letters of assent and those of their families were collected and then their demographic forms were filled in which contained data about age, gender, the beginning age of Schizophrenia, the numbers of hospitalization, education, marital status, job, disease background, psychopathic disease family background, and the kind of consumed drugs were given through interviewing the patient, his close relatives and also his doctors and nurses.

The means to collect data included:

1) Positive and negative signs of Schizophrenia were evaluated by a psychiatrist and through PANSS (Adam et al., 2012) questionnaire. This questionnaire has 30 items: 7 items for positive signs, 7 items for negative signs, and 16 items for general psychopathology) (Kay et al., 2012).
  Based on DSM-IV-TR, the variance and reliability of the test were evaluated. Alfa coefficient shows the high variance and also the resemblance among the questionnaires was between 73% and 83%. The final indicators of re-test for patients were 89%, 82%, 81%, and 77% for negative, positive, combined, and general psychopathology criteria, respectively (Kay et al., 2012).
2) BIS criteria: This is a self-rating criteria including 30 items to evaluate individual impulsiveness in which their sub-branches are achieved. A) Cognitive Impulsiveness B) Motor Impulsiveness C) Non-planning. Cognitive impulsiveness shows no endurance, no correlation and also cognitive boredom. Motor impulsiveness shows acting with no thinking and Non-planning represents the lack of planning for future. Higher scores are the signs for the higher levels of impulsive behavior (Patton et al., 1995&Javid et al., 2012).
3) Patient’s function was evaluated at the acute phase and according to GAF: DSM –IV-TR (B. J. Sadock & V. A. Sadock, 2007).
4) Drugs´ side effects were evaluated based on the AIMS questionnaires (B. J. Sadock & V. A. Sadock, 2007). The results were reported as average-standard deviation.
  Cigarette and drug addictions were defined based on DSM-IV-TR Scale. Cigarette consumption for 20 Cigarette a day and for one whole year was defined as nicotine addiction. The statistical analysis was done with Spss-16. For the qualitative and quantitative variables, Chi-square and t-test were used, respectively. The data correlation was done, value <0.05 was considered as the meaningful statistical level.

## 3. Results

The patients´ age average was 36.77 ±9.6, ranging from 18 to 63. Of 100 patients, 79 were male. 53 of the whole patients (53%) mentioned some psychiatric cases in their families. The average age to diagnose Schizophrenia was 24.64 ±6.7. Demographic data were showed in Tables 1 and 2.

**Table 1:** Demographic data of patients with & without smoking

Schizophrenic patients	Smoking (+)	Smoking (-)	P-Value
**Age**	38.20±9.5	35.34±9.6	0.1
**Gender**			0.001
**Men**	100%	58%	
**Women**	0	42%	
**Marriage (%)**			0.056
**Single**	58%	76%	
**Married**	42%	24%	
**Family History**			0.3
**No**	26%	21%	
**Yes**	24%	29%	
**Education**			0.001
**High School Degree**	44%	29%	
**High School Degree and Upper**	6%	21%	
**Substance Abuse**			0.001
**No**	20%	48%	
**Yes**	30%	2%	
**First Age of Diagnosis**	25.9±7.1	22.9±6.7	0.03
**Intervention Duration**	12.3±9.2	12.48±8.4	0.9
**First Age of Hospitalization**	29.6±9.8	25.5±7.5	0.1
**Number of Hospitalization**	4.3±4.4	5.06±4.4	0.1

**BMI**	**22±3.3**	**24.2±5.4**	**0.04**

**Table 2:** Demographic data of patients with & without substance abuse

Schizophrenic patients	Substance abuse (+)	Substance abuse (-)	P-Value
**Age**	35.19±9.6	37.51±9.6	0.2
**Gender**			0.001
**Men**	100%	59.5%	
**Women**	0	40.5%	
**Marriage (%)**			0.5
**Single**	65.6%	67.6%	
**Married**	35.4%	32.4%	
**Family History**			0.2
**No**	18%	29%	
**Yes**	14%	39%	
**Education**			0.006
**Under diploma**	19%	44%	
**Diploma and Upper**	3%	24%	
**First Age of Diagnosis**	27.38±7.1	23.06±6.6	0.004
**Intervention duration**	7.81±6.8	14.57±8.9	0.001
**First Age of Hospitalization**	31.00±9.6	26.03±8.2	0.008
**Number of Hospitalization**	2.88±2.6	5.57±4.8	0.004

**BMI**	**21.59±4.0**	**23.48±4.7**	**0.006**

Addiction abundance of cigarette was 50% which belonged to men. Addiction abundance of drugs was 32%, belonging to men. 25 of them consumed some dugs simultaneously, 6 of them just consumed opium and in one case just Meta-amphetamines was consumed. 94 of patients took atypical antipsychotic drugs and 6 of them took typical antipsychotic drugs. Cigarette addiction came with conceptual disorganization and stereotyped thinking, while PANSS and drug addiction came with delusion, hallucinatory behavior and the lack of spontaneity and flow of conversation, PANSS.

There was a connection between gender and cigarette and drug addictions (a=0.0001) and it was common in men. Education and cigarette and drug addiction were related to each other. About cigarette addiction it was a=0.001 and about drugs it was a=0.006 and among people with lower education it seemed more. There was no connection between addiction and marital status. About cigarette it was a=0.08 and for drugs a=0.8. There was no connection between psychiatric family background and cigarette addiction a=0.3 and drug addiction a=0.2. There was no meaningful statistical difference between the number of hospitalization of addicted patients and healthy people.

Positive PANSS score has a meaningful statistical relation with drug and cigarette consumption (p-value=0.001). BARAT had a correlation with P_3_ (a=0.008, r=0.2) P_1_ (a=0.04, r=0.1) N_7_ (a=0.01, r=0.2) N_2_ (A=0.003, R=0.2). Cigarette addiction had a correlation with BARAT score (P-Value =0.005, a=0.005), but there was no meaningful statistical relation between BARAT and drug consumption. Cigarette consumption had a direct correlation with sub-components of impulsive behavior, sudden behaviors, while (r=0.3), (a=0.001) and movement. On the other hand, drug consumption had a correlation with the sub-components of movement sudden behaviors (r=0.2, a=0.01). Of the variables having a correlation with drug consumption, just cigarette factor and treatment period in a regression model could predict drug consumption (Tables 3 & 4).

**Table 3:** Relation between psychopathologic variables with smoking

Schizophrenic patients	Smoking (+)	Smoking (-)	P-Value
**BARAT**	81.89±10.6	23.9±6.01	0.005
**Motor impulsiveness**	28.7±5.3	30.9±3.7	0.001
**Nonplanning**	31.1±3.2	19.5±4.9	0.7
**Cognitive impulsiveness**	21.9±4.6	3.1±1.3	0.01
**PANSS**	116.4±12.9	112.4±14.7	0.1
**Positive Symptoms Score**	31.8±4.1	28.5±5.2	0.001
**Negative Symptoms Score**	26.3±6.04	26.1±6.2	0.8
**General Psychopathology Score**	58.5±7.5	57.9±8.2	0.7
**Conceptual disorganization**	4.2±1.4	3.5±1.6	0.03
**Stereotyped thinking**	**4.2±0.7**	**3.7±1.02**	**0.01**

**Table 4:** Relation between psychopathologic variables and substance abuse

Schizophrenic patients	Substance abuse (+)	Substance abuse (-)	P-Value
**BARAT**	81.4±11.0	76.5±12.7	0.1
**Motor impulsiveness**	28.7±5.4	25.2±6.2	0.008
**Nonplanning**	30.8±3.5	31.1±3.5	0.7
**Cognitive impulsiveness**	21.8±4.6	20.2±5.0	0.01
**PANSS**	116.2±12.1	113.6±14.7	0.3
**Positive Symptoms Score**	32.1±4.2	29.2±5.0	0.006
**Negative Symptoms Score**	25.7±5.5	26.4±6.3	0.5
**General Psychopathology Score**	58.5±7.1	58.1±8.2	0.7
**AIMS**	0.5±1.1	0.7±2.3	0.4
**Delusions**	5.2±1.2	4.6±1.3	0.04
**Hallucinatory behavior**	4.5±1.7	3.7±1.7	0.03
**Lack of spontaneity and flow of conversation**	**2.2±1.2**	**3.0±1.3**	**0.01**

## 4. Discussion

According to the performed studies, this is among the rare studies done about patients in Iran with Schizophrenia considering cigarette and drug consumption and their relation with impulsiveness and while focusing on positive and negative syndrome of Schizophrenia with their demographic characteristics.

In this study all patients are the ones with chronic Schizophrenia which is one of the good features of it. In our study the number of hospitalization in patients with drug consumption record was lower than that of patients with no drug consumption record. Bunette (2006) did a study an opposite direction.

Cigarette consumption prevalence in these patients was 50% which seems to be lower than the reported percent in patients with Schizophrenia based on the previous Figures equaling 85 -95% (Karşıdag et al., 2005) and in the study done in Iran, it was 71-98% (Fazel et al., 2009). This statistical difference can be due to the difference in case volume, specifically the disease period, cultural matters and the availability of drugs in Iran and further its distribution in different provinces. As the other determining factor, cigarette is used for self-treatment to reduce the side-effects of anti-psychotics (Kumari et al., 2005) and it should be noted that these patients used Atypic anti-psychotics whose extrapyramidal effects are less than those of typicanti-psychotics.

In this study, there was no meaningful relation between PANSS score and cigarette, drug consumption which showed there is a positive and negative score in patients with Schizophrenia who smoke cigarette (Adam et al., 2012). This difference in results can be related to disease stage, the reduction in Schizophrenia symptoms due to the passing time and the proportional stabilization of patients´ clinical condition (First, Gibbon, Spitzer, Williams, & Benjamin, 1997).

High score in cognitive impulsiveness, movment sudden behavior of BARAT questionnaire in patients addicted to cigarette and moving sudden behavior in drug consumption were seen which were based on the previous studies (Adam et al., 2012).

High score in cognitive impulsiveness and movement sudden behavior in this study can be a predictive factor for the probability of more impulsive behaviors like suicide which is in great need of more attention to Co-morbidities of cigarette and drug addiction in these patients.

## 5. Conclusion

Easy and available sampling was used and the study was cross-sectional through which we find that there is no relation between disease and drug consumption.

As the results are based on the studies done on hospitalized patients, it is not possible to generalize the results for all patients with chronic Schizophrenia.

Patients with chronic Schizophrenia were studied, but the sickness period and its effect on symptoms were not included. The good point is that the study was done on a homogeneous group of patients with Schizophrenia.

Our studies show that cigarette consumption prevalence is high in patients with Schizophrenia. Opposite of other studies, there was no relation found between the severity of psychopathology and cigarette/drug consumption. There was a relation between cigarette and impulsiveness in patients with Schizophrenia and it seems cigarette consumption is the predicator of more impulsiveness and higher impulsiveness will probably increase the rate of suicide.
